# Risk Factors of Pre-Eclampsia/Eclampsia and Its Adverse Outcomes in Low- and Middle-Income Countries: A WHO Secondary Analysis

**DOI:** 10.1371/journal.pone.0091198

**Published:** 2014-03-21

**Authors:** Ver Luanni Bilano, Erika Ota, Togoobaatar Ganchimeg, Rintaro Mori, João Paulo Souza

**Affiliations:** 1 Department of Global Health Policy, Graduate School of Medicine, The University of Tokyo, Tokyo, Japan; 2 Department of Health Policy, National Center for Child Health and Development, Tokyo, Japan; 3 Department of Reproductive Health and Research, World Health Organization, Geneva, Switzerland; University of Tennessee Health Science Center, United States of America

## Abstract

**Background:**

Pre-eclampsia has an immense adverse impact on maternal and perinatal health especially in low- and middle-income settings. We aimed to estimate the associations between pre-eclampsia/eclampsia and its risk factors, and adverse maternal and perinatal outcomes.

**Methods:**

We performed a secondary analysis of the WHO Global Survey on Maternal and Perinatal Health. The survey was a multi-country, facility-based cross-sectional study. A global sample consisting of 24 countries from three regions and 373 health facilities was obtained via a stratified multi-stage cluster sampling design. Maternal and offspring data were extracted from records using standardized questionnaires. Multi-level logistic regression modelling was conducted with random effects at the individual, facility and country levels.

**Results:**

Data for 276,388 mothers and their infants was analysed. The prevalence of pre-eclampsia/eclampsia in the study population was 10,754 (4%). At the individual level, sociodemographic characteristics of maternal age ≥30 years and low educational attainment were significantly associated with higher risk of pre-eclampsia/eclampsia. As for clinical and obstetric variables, high body mass index (BMI), nulliparity (AOR: 2.04; 95%CI 1.92–2.16), absence of antenatal care (AOR: 1.41; 95%CI 1.26–1.57), chronic hypertension (AOR: 7.75; 95%CI 6.77–8.87), gestational diabetes (AOR: 2.00; 95%CI 1.63–2.45), cardiac or renal disease (AOR: 2.38; 95%CI 1.86–3.05), pyelonephritis or urinary tract infection (AOR: 1.13; 95%CI 1.03–1.24) and severe anemia (AOR: 2.98; 95%CI 2.47–3.61) were found to be significant risk factors, while having >8 visits of antenatal care was protective (AOR: 0.90; 95%CI 0.83–0.98). Pre-eclampsia/eclampsia was found to be a significant risk factor for maternal death, perinatal death, preterm birth and low birthweight.

**Conclusion:**

Chronic hypertension, obesity and severe anemia were the highest risk factors of preeclampsia/eclampsia. Implementation of effective interventions prioritizing risk factors, provision of quality health services during pre-pregnancy and during pregnancy for joint efforts in the areas of maternal health are recommended.

## Introduction

Pre-eclampsia has an immense adverse impact on maternal and perinatal health, especially in the developing world. It is a major cause of almost a third of a million maternal deaths in low- and middle-income settings [1], [2] and also accounts for substantial proportions of the more than six million perinatal deaths [3], approximately eight million preterm births [4] and almost 20 million low birthweight infants in developing nations [5]. Furthermore, pre-eclampsia and its adverse outcomes have been linked to higher risks of chronic non-communicable diseases (NCDs) in later life, thereby posing a daunting challenge within the context of double burden and limited resources in the developing world [6].

Since pre-eclampsia's etiology remains unknown [7], investigation and identification of the most important risk factors is vital for policy and clinical purposes including prioritization of interventions, resource allocation, identification of high-risk pregnant women for more intensive observation and care, and development or improvement of risk management strategies. While most studies have been undertaken in high-income settings, some inconsistencies exist (e.g. whether or not maternal education is a significant risk factor), especially in developing settings where pre-eclampsia risk factors have been explored less. Furthermore, other conditions such as maternal infections, severe anemia and lack of antenatal care that are more prevalent in less developed regions require further investigation and validation of findings [8]–[10]. In addition, previous research has been limited by small sample sizes [11] or analytic methods that do not properly take into account the effects of higher-level factors [12], [13].

As for pre-eclampsia's adverse maternal and perinatal outcomes, there is limited information and research assessing the magnitude of risks in low-resource areas where the impact is thought to be more severe [14]. Furthermore, small sample sizes or lack of adjustment for some important confounders are notable weaknesses that have restricted previous research [11], [15].

This study thus aimed to conduct multi-level analyses of data from the WHO Global Survey on Maternal and Perinatal Health including 23 developing countries in Africa, Latin America and Asia in order to estimate associations between maternal, country characteristics and pre-eclampsia/eclampsia, and to estimate the magnitude of risks for pre-eclampsia/eclampsia's adverse maternal and perinatal outcomes.

## Methods

### Ethics Statement

The protocol was approved by the WHO's Scientific and Ethical Review Group and Ethics Review Committee and that of each country independently [16]. Written consent was obtained from each participating country's ministry of health and each selected facility's director [16]. Because the study involved cluster-level inclusion and records data extraction without individual identifying information, individual informed consent was not obtained.

### Study design

We undertook a secondary analysis of the WHO Global Survey on Maternal and Perinatal Health. Details of this survey's methodology are available elsewhere [16]. To summarize, the survey was a multi-country, facility-based cross-sectional study. It was conducted between 2004 and 2005 in Africa and Latin America, and between 2007 and 2008 in Asia. A global sample of countries and health facilities was obtained via a stratified multistage cluster sampling design. Twenty-four countries from three regions were included in the survey: Algeria, Angola, Democratic Republic of Congo, Niger, Nigeria, Kenya, and Uganda from Africa; Argentina, Brazil, Cuba, Ecuador, Mexico, Nicaragua, Paraguay, and Peru from Latin America; and Cambodia, China, India, Japan, Nepal, Philippines, Sri Lanka, Thailand, and Vietnam from Asia. For each country, the capital city and two randomly selected provinces were included in the sample. Only health facilities with caesarean section (C-section) capacities and with reported annual deliveries of at least 1,000 were eligible for inclusion. Up to seven facilities per country were randomly selected. A total of 373 facilities were included in the survey. The recruitment and data collection period varied depending on the volume of deliveries in the sampled facilities. Specifically, it was set for three months for facilities with up to 6,000 annual deliveries and two months for those with more than 6,000 annual deliveries. All pregnant women admitted for delivery during the recruitment period were included.

### Data Sources

Data for individuals and institutions were sourced from the WHO Global Survey on Maternal and Perinatal Health. Individual data were obtained via direct extraction from patient records by trained medical personnel within seven days after delivery or before maternal hospital discharge. Institutional data were collected using a standard form by hospital coordinators in consultation with the director or head of obstetrics, and country information was obtained from the World Bank [17] and literature [18].

### Main Outcomes Variable Definitions

Due to secondary data limitations and based on previous research [13] pre-eclampsia and its complication, eclampsia, were combined into a single outcome variable. Pre-eclampsia was defined as high blood pressure (≥140 mmHg systolic or ≥90 mmHg diastolic, or increases of 30 mmHg systolic or 15 mmHg diastolic from the baseline on at least two occasions six or more hours apart) that develops from the 20^th^ gestational week in a previously normotensive woman, and proteinuria. The outcome can be based on having the recorded diagnosis or on blood pressure and urine test findings, even without specifying the condition. Eclampsia was defined as the onset of convulsions described as grand-mal type seizures first appearing before or during labour, or within 48 hours from delivery, and/or coma unrelated to other cerebral conditions in women with pre-eclamptic signs and symptoms.

Adverse maternal and perinatal outcomes including maternal death, perinatal death, preterm birth and low birthweight were investigated. Maternal death was defined as intrahospital death of a woman that occurred on or before the 8^th^ day postpartum. Perinatal death was defined as early neonatal death (i.e. intrahospital death of an infant that occurred on or before the 7^th^ day after delivery) or stillbirth (fresh or macerated). Preterm birth was defined as livebirth occurring at less than 37 completed gestational weeks and low birthweight was defined as birthweight <2500 grams of a liveborn infant regardless of gestational age.

### Independent Variables Definitions

The individual characteristics investigated were: maternal age, marital status, maternal educational level, maternal body mass index (BMI), parity, maternal clinical conditions (chronic hypertension, gestational diabetes, cardiac/renal disease, pyelonephritis or urinary infection, severe anemia) and number of antenatal visits. Maternal age was defined as age in completed years at the time of delivery. Participants were classified into four categories: <20, 20 to 29, 30 to 34 and ≥35 years. Marital status was dichotomized into single/widowed/divorced/separated and married/cohabiting with the infant's father. Maternal education was based on the number of years of schooling. The UNESCO international standard classification of education was adopted allocating participants to one of five levels: no education (0 years), primary (1 to 6 years), lower secondary (7 to 9 years), upper secondary (10 to 12 years) and post-secondary/tertiary (>12 years).

Maternal BMI was computed as the ratio of maternal weight in kilograms and the square of maternal height in meters. In Africa and Latin America, maternal weight was recorded at the last antenatal care visit, while in Asia, it was recorded at admission for delivery. Maternal BMI was categorized into four ranges: <20, 20 to <26, 26 to <35 and ≥35 kg/m^2^. Parity was defined as the number of previous births and was dichotomized into nulliparity and multiparity. Maternal clinical conditions were determined based on the recorded diagnosis or laboratory findings, even without specifying the condition. Chronic hypertension was defined as high blood pressure diagnosed prior to the onset of pregnancy or before the 20^th^ gestational week. Severe anemia was defined as a haemoglobin level of <7 g/dL during pregnancy. The number of antenatal visits was irrespective of whether the antenatal clinic was different from the admission facility. This was categorized into four based on WHO recommendations for antenatal care: 0, 1 to 3, 4 to 8 and >8 visits.

### Institutional Characteristics

We considered institutional information related to maternal and perinatal care, such as: availability of laboratory tests; anesthesiology resources; services for intrapartum care, delivery and newborn care; and the presence of basic emergency medical and obstetric care facilities, intensive care units, and training resources. As with previous research using the WHO Global Survey data, we applied institutional capacity index scoring to gauge facilities' levels of resources as well as their maternal and perinatal care service provision capabilities. Points were assigned for the availability of resources or services and the unweighted total was obtained. We then dichotomized the institutional capacity index score into higher capacity (greater than the mean for all facilities) and lower capacity (less than or equal to the mean for all facilities).

### Country Characteristics

The country characteristics considered were gross national income (GNI) per capita and maternal mortality ratio (MMR). We categorized GNI per capita from the World Development Indicators according to World Bank definitions of low income ($935 or less), lower-middle income ($936 to $3,705) and upper-middle income ($3,706 to $11,455). MMRs (maternal deaths per 100,000 livebirths) were obtained from and followed *The Lancet* series on maternal and child mortality's categorization of low (below 100), moderate (100 to 299), high (300 to 549) and very high (550 and above) [18].

### Study Population

The study population for the secondary analyses included women with singleton neonates of at least 20 weeks of gestation. Those with missing information on the inclusion criteria and on the pre-eclampsia outcome were excluded. Japan was also excluded due to the study's focus on developing settings.

### Statistical Analysis

Statistical analyses were conducted using Stata/MP version 12.1 (Stata Corp LP, College Station, Texas). Descriptive statistics (i.e. proportions) to examine the distribution of the outcomes and independent variables were generated. Univariate logistic regression analyses were also conducted for initial inspection of the associations between the outcomes and independent variables. For the main objectives, multi-level multiple regression modelling was applied. In general, this involved three levels corresponding to the individual, institutional and country characteristics with random effects at the facility and country levels to account for unmeasured higher-level factors and for clustering due to the sampling design. Significance testing for random effects was conducted via likelihood ratio tests which involved comparing nested models with models that included additional higher levels. A p-value of <0.05 was considered statistically significant. For the associations between pre-eclampsia/eclampsia and the individual, institutional and country characteristics, a single logistic model was employed to simultaneously estimate the relationship of the independent variables with the outcome. To estimate associations of pre-eclampsia/eclampsia with adverse maternal and perinatal outcomes, we created separate logistic regression models for each of the adverse outcomes and adjusted for important confounders identified from literature and the results of the previous risk factor analysis. Two types of sensitivity analyses were conducted for all models to address missing BMI information (>10% for a number of countries) by: a) including a missing category for BMI and b) excluding countries with more than 10% of BMI data missing, namely: Kenya (86%), Brazil (67%), Angola (40%), Argentina (32%), Uganda (22%), Philippines (20%), and Peru (13%).

## Results

The WHO Global Survey on Maternal and Perinatal Health dataset included a total of 290,610 deliveries. For this secondary analysis, data for 276,388 mothers and their infants was retained after excluding Japan, multiple births, deliveries at less than 20 weeks of gestation and those with missing information on the inclusion criteria and pre-eclampsia/eclampsia outcome. Overall and regional profiles for the study population are provided in Table 1. The majority of mothers were aged between 20 to 29 years old (59%), were married or cohabiting with the infant's father (86%) or had given birth previously (57%). As for education and BMI, the upper secondary level (37%) and the 20 to <26 kg/m^2^ (48%) category had the greatest proportions of mothers respectively. At the facility level, the majority (65%) of deliveries was in institutions with higher capacity index scores except for Africa, where a greater portion (63%) of mothers delivered in lower-capacity facilities. At the country level, the greatest proportions were in the lower-middle GNI per capita category (49%) and the low MMR level (60%) overall, but these characteristics varied across regions.

**Table 1:** Overall and regional distribution of risk factors for pre-eclampsia/eclampsia.

Characteristic	Deliveries		Pre-eclampsia/eclampsia	
	N	%	n	%
**Individual**				
Maternal age				
<20	34,824	12.6	1,491	4.3
20 to 29	162,466	58.9	5,531	3.4
30 to 34	49,117	17.8	2,022	4.1
> = 35	29,461	10.7	1,696	5.8
Marital status				
Single/divorced/separated/widowed/other	37,356	13.6	1,945	5.2
Married/cohabiting	238,193	86.4	8,764	3.7
Education				
None	20,735	8.8	602	2.9
Primary	54,813	23.4	2,345	4.3
Lower secondary	35,370	15.1	1,276	3.6
Upper secondary	87,574	37.4	3,551	4.0
Post-secondary/tertiary	35,881	15.3	1,462	4.1
BMI (kg/m^2^)				
<20	7,707	3.3	156	2.0
20 to <26	111,049	47.7	2,803	2.5
26 to <35	105,905	45.5	4,737	4.5
> = 35	8,221	3.5	888	10.8
Parity				
Nulliparous	118,348	43.0	5,527	4.7
Multiparous	156,589	57.0	5,178	3.3
Chronic hypertension				
Yes	2,334	0.8	653	28.0
No	273,769	99.2	10,093	3.7
Gestational diabetes				
Yes	1,940	0.7	204	10.5
No	274,163	99.3	10,544	3.8
Cardiac/renal disease				
Yes	1,254	0.4	151	12.0
No	274,848	99.6	10,597	3.9
Pyelonephritis/urinary tract infection				
Yes	18,230	6.6	1,169	6.4
No	257,825	93.4	9,574	3.7
Severe anemia				
Yes	1,529	0.6	192	12.6
No	274,555	99.4	10,555	3.8
Antenatal care visits				
0	12,750	5.2	689	5.4
1 to 3	67,124	27.4	2,217	3.3
4 to 8	127,162	52.0	5,088	4.0
>8	37,658	15.4	1,556	4.1
**Institutional**				
Facility capacity				
Lower	96,723	35.0	2,112	2.2
Higher	179,665	65.0	8,642	4.8
**Country**				
GNI per capita				
Low	83,915	30.4	2,045	2.4
Lower-middle	134,280	48.6	5,185	3.9
Upper-middle	58,193	21.0	3,524	6.1
Maternal mortality ratio				
Low	166,660	60.3	7,272	4.4
Moderate	47,117	17.0	1,880	4.0
High	57,217	20.7	1,556	2.7
Very high	5,394	2.0	46	0.8

**Figure 1:**
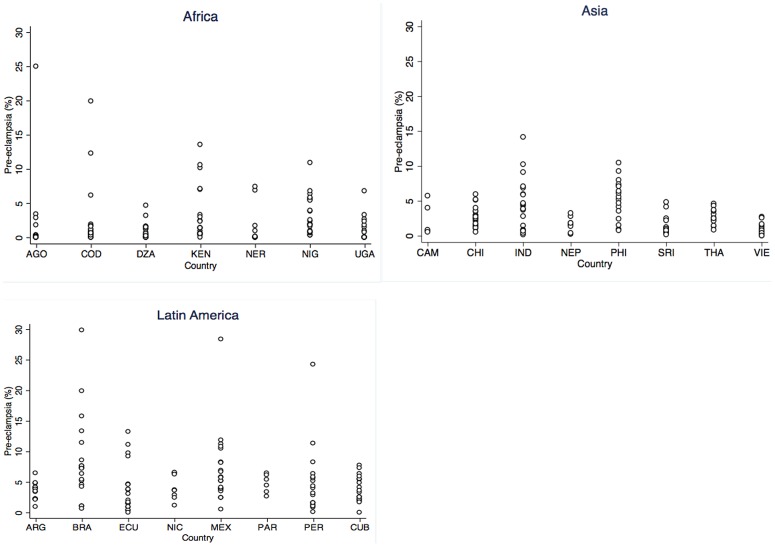
Pre-eclampsia prevalence per facility by country and region. Plot showing prevalence of pre-eclampsia per facility by country and region (Africa, Asia and Latin America).

The prevalence of pre-eclampsia/eclampsia in the study population was 10,754 (4%), but there was much variation between and within countries. Regional, country and facility pre-eclampsia/eclampsia prevalence profiles are provided in Table 2 and Figure 1. Across regions, pre-eclampsia/eclampsia prevalences at the country level ranged from less than 1% in Angola to 8% in Brazil. Such variability was also observed among countries within the same region. The highest maternal mortality was observed in Nigeria at 8 (3%), and the highest perinatal mortality was in the Democratic Republic of Congo at 44 (22%) associated with pre-eclampsia/eclampsia. The highest prevalence of preterm birth was Angola at 35 (76%) and the highest rate of low birthweight was in Sri Lanka at 83 (40%) associated with pre-eclampsia/eclampsia.

**Table 2:** Distribution of pre-eclampsia and its adverse maternal and perinatal outcomes in the study population by region and country.

Region	Country	All Deliveries	Pre-eclampsia									
			Deliveries		Maternal death		Perinatal death		Preterm birth		Low birthweigh	
		N	n	%	n	%	n	%	n	%	n	%
**Africa**		**77,884**	**1,804**	**2.32**	**25**	**1.39**	**296**	**16.46**	**469**	**26.71**	**400**	**23.92**
	Algeria	15,273	202	1.32	1	0.50	23	11.39	53	27.32	63	36.00
	Angola	5,394	46	0.85	1	2.17	8	17.39	35	76.09	10	21.74
	DRC*	8,404	199	2.37	8	4.0	44	22.11	33	17.10	35	18.13
	Kenya	18,767	506	2.70	3	0.59	99	19.6	161	32.33	158	32.58
	Niger	8,123	88	1.08	1	1.14	12	13.64	11	12.79	19	22.35
	Nigeria	8,699	271	3.12	8	2.97	53	19.56	84	31.94	54	22.41
	Uganda	13,224	492	3.72	3	0.61	57	11.70	92	19.33	31	21.38
**Latin America**		**95,296**	**5,718**	**6.00**	**3**	**0.05**	**186**	**3.26**	**1,176**	**20.96**	**1,285**	**23.56**
	Argentina	10,335	334	3.23	NR		6	1.80	93	28.01	91	28.89
	Brazil	14,625	1,203	8.23	1	0.08	35	2.94	200	16.82	242	21.30
	Cuba	12,521	477	3.81	NR		19	3.98	83	17.89	89	20.05
	Ecuador	12,310	818	6.65	1	0.12	14	1.71	97	12.28	160	20.20
	Mexico	20,712	1,510	7.29	1	0.07	51	3.38	381	25.73	364	25.26
	Nicaragua	5,599	256	4.57	NR		10	3.91	38	15.28	51	20.65
	Paraguay	3,374	166	4.92	NR		11	6.67	44	26.67	40	25.64
	Peru	15,820	954	6.03	NR		40	4.19	240	25.48	248	27.49
**Asia**		**103,208**	**3,232**	**3.13**	**22**	**0.68**	**307**	**9.48**	**868**	**27.26**	**1,090**	**34.45**
	Cambodia	5,458	155	2.84	1	0.64	20	12.82	48	31.37	52	34.67
	China	14,373	402	2.80	1	0.25	13	3.23	81	20.66	76	20.27
	India	24,421	1,123	4.60	14	1.25	177	15.76	391	35.07	444	39.71
	Nepal	8,265	180	2.18	2	1.08	20	10.75	43	24.43	69	38.12
	Philippines	13,169	738	5.60	4	0.54	60	8.13	146	20.17	244	33.56
	Sri Lanka	14,891	208	1.40	NR		10	4.81	68	32.69	83	39.90
	Thailand	9,656	272	2.82	NR		5	1.84	70	25.93	91	35.00
	Vietnam	12,975	154	1.19	NR		2	1.30	21	14.38	61	13.65

*Democratic Republic of Congo; NR- not reported.

Overall and regional profiles for pre-eclampsia/eclampsia risk factors are provided in Table 1. Table 3 shows the adjusted odds-ratios (AOR) for pre-eclampsia/eclampsia risk factors obtained via multi-level multiple logistic regression analyses. At the individual level, sociodemographic characteristics of older maternal age (≥30 years), lower than secondary education attainment such as primary level (AOR: 1.11; 95% confidence interval (CI): 1.01–1.22) or no education (AOR: 1.22; 95%CI 1.07–1.39), high BMI (≥26 kg/m^2^) (≥35 kg/m^2^, AOR: 3.90; 95%CI 3.52–4.33), nulliparity (AOR: 2.04; 95%CI 1.92–2.16), were significantly associated with higher odds of having pre-eclampsia/eclampsia. As for clinical variables, history of chronic hypertension (AOR: 7.75; 95%CI 6.77–8.87), gestational diabetes (AOR: 2.00; 95%CI 1.63–2.45), cardiac or renal disease (AOR: 2.38; 95%CI 1.86–3.05), pyelonephritis or urinary tract infection (AOR: 1.13; 95%CI 1.03–1.24) and severe anemia (AOR: 2.98; 95%CI 2.47–3.61) were also significantly associated with higher pre-eclampsia/eclampsia risks. Antenatal care non-attendance was associated with significantly higher pre-eclampsia/eclampsia risks (AOR: 1.41; 95%CI 1.26–1.57) while having >8 visits was protective (AOR: 0.90; 95%CI 0.83–0.98). Lastly at the country level, a significant increasing trend in pre-eclampsia/eclampsia odds was observed with rising GNI per capita. Similar findings were obtained from the sensitivity analyses.

**Table 3:** Adjusted odds ratios (ORs) of risk factors for pre-eclampsia/eclampsia.

Risk factor	Adjusted OR (95% CI)	p-value
**Individual**		
Maternal age		
<20	0.97 (0.89–1.05)	0.458
20 to 29	Ref	
30 to 34	1.40 (1.31–1.51)	<0.001
> = 35	1.95 (1.80–2.12)	<0.001
Marital status		
Single/divorced/separated/widowed/other	0.98 (0.90–1.06)	0.577
Married/cohabiting	Ref	
Education		
None	1.22 (1.07–1.39)	0.003
Primary	1.11 (1.01–1.22)	0.033
Lower secondary	1.05 (0.95–1.17)	0.347
Upper secondary	1.06 (0.98–1.15)	0.172
Post-secondary/tertiary	Ref	
BMI (kg/m^2^)		
<20	0.86 (0.72–1.03)	0.105
20 to <26	Ref	
26 to <35	1.71 (1.61–1.81)	<0.001
> = 35	3.90 (3.52–4.33)	<0.001
Parity		
Nulliparous	2.04 (1.92–2.16)	<0.001
Multiparous	Ref	
History of chronic hypertension		
Yes	7.75 (6.77–8.87)	<0.001
No	Ref	
Gestational diabetes		
Yes	2.00 (1.63–2.45)	<0.001
No	Ref	
Cardiac/renal disease		
Yes	2.38 (1.86–3.05)	<0.001
No	Ref	
Pyelonephritis/urinary tract infection		
Yes	1.13 (1.03–1.24)	0.007
No	Ref	
Severe anemia		
Yes	2.98 (2.47–3.61)	<0.001
No	Ref	
Antenatal care visits		
0	1.41 (1.26–1.57)	<0.001
1 to 3	1.01 (0.94–1.08)	0.752
4 to 8	Ref	
>8	0.90 (0.83–0.98)	0.017
**Institutional**		
Facility capacity		
Lower	0.47 (0.37–0.60)	<0.001
Higher	Ref	
**Country**		
GNI per capita		
Low	Ref	
Lower-middle	2.04 (1.03–4.02)	0.040
Upper-middle	3.55 (1.57–8.02)	0.002
Maternal mortality ratio		
Low	Ref	
Moderate	1.97 (1.11–3.48)	0.020
High	1.73 (0.78–3.84)	0.180
Very high	0.50 (0.16–1.55)	0.231

Three-level structure logistic random effects regression models were used to obtain the Adjusted ORs: individual (level 1); facility (level 2); and country (level 3) adjusted with variables in the table.

For adverse maternal and perinatal outcomes, overall prevalences were less than 335 (1%) for maternal death, 7,404 (3%) for perinatal death, 27,611 (10%) for preterm birth and 27,348 (10%) for low birthweight. Prevalences of the adverse outcomes by region and country are given in Table 2. Table 4 provides the adjusted odds-ratios for adverse maternal and perinatal outcomes obtained via multi-level logistic regression analyses. Random effects for the institutional and country levels in each model were shown to be significant from likelihood-ratio tests. For maternal death (AOR: 4.48; 95%CI 2.99–6.69) and perinatal death (AOR: 1.87; 95%CI 1.66–2.11), preterm birth (AOR: 2.86; 95%CI 2.68–3.06) and low birthiweight (AOR: 2.32; 95%CI 2.16–2.50), pre-eclampsia/eclampsia was found to be a significant risk factor. Similar findings were obtained from the sensitivity analyses.

**Table 4:** Adjusted odds ratios (ORs) for adverse maternal and perinatal outcomes of pre-eclampsia.

Adverse outcome	Adjusted OR (95% CI)	p-value
Maternal death	4.48 (2.99–6.69)	<0.001
Perinatal death^a^^, ^ b	1.87 (1.66–2.11)	<0.001
Preterm birth^a^	2.86 (2.68–3.06)	<0.001
Low birthweight^a^^, ^ b	2.32 (2.16–2.50)	<0.001

Adjusted for facility capacity index score at facility level, GNI per capita and maternal mortality ratio at the country level, marital status, maternal education, maternal age, maternal BMI, parity, clinical conditions (hypertension, diabetes, cardiac/renal disease, pyelonephritis or urinary infection, severe anemia) and number of antenatal care visits at the individual level.

a Also adjusted for infant sex.

b Also adjusted for gestational age.

## Discussion

To the authors' knowledge, this is the first multi-level, multi-country analyses dealing with pre-eclampsia/eclampsia risk factors and its adverse outcomes including maternal deaths focusing on developing settings. Also among the strengths of this study are the large sample size and the application of standardized questionnaires across facilities, countries and regions. The prevalences of pre-eclampsia/eclampsia in the study population showed considerable variation at both the country and facility levels. At the individual level, a history of chronic hypertension, a BMI ≥35 kg/m^2^, and severe anemia significantly increased the risk of pre-eclampsia/eclampsia by three times or more. Other significant higher maternal risk factors (twice the odds or greater) included having cardiac or renal disease, having diabetes, being nulliparous and being ≥30 years of age. Mothers diagnosed with pre-eclampsia/eclampsia were found to be significantly at four times the risk of maternal death than mothers who did not have pre-eclampsia/eclampsia. Our analysis found that protective factors for preeclampsia/eclampsia included >8 antenatal care visits compared with 4–8 visits. The MMR was found to be significantly associated with pre-eclampsia/eclampsia risk factors. The condition was also shown to have a higher risk for perinatal death, preterm birth and low birth weight. These findings support previous research [3], [4].

The variation of pre-eclampsia/eclampsia prevalences is perhaps not only reflective of the variability in maternal risk-factor distribution, but may also be attributed to differences in facility and country characteristics, such as diagnostic capacities or accessibility of services. While the facility's referral level is also a factor, considerable variability was still observed even after stratification on this variable. The above observations are supported by findings that GNI per capita are significant pre-eclampsia/eclampsia risk factors. This may be explained by countries with lower GNI per capita having less diagnostic capabilities to identify pre-eclampsia/eclampsia cases. Lower GNI per capita may also be reflective of socioeconomic factors associated with poor access to healthcare services. These findings suggest probable underestimation of pre-eclampsia/eclampsia prevalences in low-resource settings as well as failure to identify high-risk pregnant women for proper management, potentially leading to increased incidence of adverse outcomes. Implications thus include improvement in diagnostic capabilities and in accessibility to healthcare services. Additionally, the significant association of moderate maternal mortality with higher pre-eclampsia/eclampsia risk compared to low maternal mortality may be reflective of other factors that have not been explored in this study and warrant further investigation.

The three highest risk factors for pre-eclampsia/eclampsia at the individual level were a history of chronic hypertension, high maternal BMI and severe anemia. Other risk factors included the clinical conditions of having cardiac or renal disease and having diabetes as well as obstetric characteristics of nulliparity and older maternal age. The above findings corroborate and add to previous research conducted in developed and in developing settings [11], [13], [19]. The associations between these risk factors and pre-eclampsia/eclampsia may be explained by various proposed pathophysiologic pathways. For the interrelated conditions of high BMI, hypertension and diabetes, the accompanying insulin resistance and hypertriglyceridemia have been suggested to contribute to the endothelial dysfunction linked to pre-eclampsia incidence [11], [13]. A systematic review conducted in 2005 [19] assessed controlled studies published over the period 1966–2002 and aimed to determine the risk of pre-eclampsia associated with factors that may be detected at antenatal check-ups. The review did not include severe anemia as a risk factor, despite the inclusion of a higher BMI and a history of pre-eclampsia [19]. A retrospective case-control study at eastern Sudan's Kassala hospital is the only study to show that women with severe anemia (<7 g/dl) had a 3.6 times significantly higher risk of pre-eclampsia compared to women with no anemia [8]. This study was based on data from only one hospital, whereas our study is the first to confirm the association between severe anemia and pre-eclampsia/eclampsia using a large global dataset.The associated micronutrient and antioxidant deficiencies are probable contributors to the development of pre-eclampsia/eclampsia. For the obstetric risk factors of nulliparity and older maternal age, several hypotheses involving maternal immune maladaptation [11], [13], and aging-mediated vascular damage [11], [13], [19] have been proposed respectively. Since all the above risk factors can be identified during antenatal check-ups, risk screening and the referral of higher-risk pregnancies for more intensive care at the check-up stage could help reduce the incidence of pre-eclampsia/eclampsia or its adverse outcomes. These corroborate hypotheses from previous research in developing settings proposing achievement of pre-eclampsia risk reduction via provision of opportune and effective antenatal care through a well-functioning health system [20], [21]. This then necessitates improving the availability, accessibility and quality of maternal healthcare facilities and services, thus reiterating the call to strengthen primary health care and health systems. Moreover, the risks posed by hypertension, high BMI, and diabetes calls attention to the less-recognized interaction of such conditions with maternal health [6]. Given that pre-eclampsia/eclampsia has been linked to increased risks of future chronic diseases as well and with the growing burden of non-communicable diseases (NCDs) in developing settings [6] joint efforts that encompass the areas of maternal health and NCDs should be explored, especially in settings with limited resources.

Additional significant risk factors were antenatal care non-attendance, attaining less than a secondary level of education and having pyelonephritis or urinary tract infection. These findings support some previous research [19], [22] but disagree with others, especially regarding education as a significant risk factor [11], [13]. The increased risks of having pre-eclampsia/eclampsia with no antenatal care attendance may be explained by inadequate management during pregnancy to prevent development of the condition. Low educational attainment on the other hand, may be indirectly representative of low socioeconomic status and its associated socioeconomic determinants, which could contribute to pre-eclampsia development [23]. Poor access to care, for instance, could lead to inadequate prenatal management and ultimately pre-eclampsia/eclampsia. As for risks associated with pyelonephritis or urinary tract infection, there are various hypotheses involving cardiovascular pathways and inflammatory responses among others. The risks posed by lack of antenatal care and a low level of education call attention to the social determinants of maternal health and reiterate the need to improve availability, utilization and quality of maternal healthcare facilities and services. On the other hand, maternal infection as a risk factor of pre-eclampsia/eclampsia requires further exploration and validation. The above findings also suggest that maternal health should not be viewed as a separate issue, but that it should be incorporated in a more comprehensive perspective and approach, especially in the context of double burden in low-resource settings [6].

This study has several limitations. First, being facility-based, the findings may not be generalizable to the broader population, especially in settings where institutional delivery rates are low. Second, there are potential biases and errors arising from the dataset. Although efforts were undertaken to standardize the data, logistical considerations and differences in medical protocols or clinical practices in the various facilities and settings precluded complete standardization. Also, because the study was not originally designed to detect pre-eclampsia/eclampsia and its outcomes or to screen for risk factors, underdiagnoses or likelier detection of more clinically evident cases could have transpired. On the other hand, missing BMI information could have biased our estimates, but the similarity of results from several sensitivity analyses suggests that this is probably not a major limitation. However, the BMI variable could contain heterogenous information because of the inconsistent timing of weight measurements in different settings. Furthermore, residual confounding probably exists in our estimates, especially with regards to the effects of smoking. Maternal smoking status could not be controlled for due to the absence of data, although it should be noted that previous research has found smoking to be a protective factor for pre-eclampsia/eclampsia and thus the magnitude of association from our study is probably biased towards the null. Lastly, perinatal mortality in our study only includes intrahospital deaths, and the inability to capture post-discharge mortality implies that our results could be underestimated. Interpretation of results should thus consider all the above limitations.

## Conclusions

In low- and middle-income settings, pre-eclampsia/eclampsia is significantly associated with maternal death, perinatal death, preterm birth and low birthweight. At the individual level, a number of sociodemographic and medical variables are significant risk factors for pre-eclampsia/eclampsia, with chronic hypertension, obesity and severe anemia posing the highest risks of the outcome, and with >8 antenatal care visits acting as a protective factor. Implementation of effective interventions targeting risk factors, provision of quality health services and joint efforts in the areas of maternal health are recommended. Prevention, early detection and timely management of pre-eclampsia/eclampsia and its risk factors at antenatal care visits have the potential to deliver considerable improvement in maternal and perinatal health in low- and middle-income settings.
